# The impact of skin colour on human photobiological responses

**DOI:** 10.1111/pcmr.12511

**Published:** 2016-08-16

**Authors:** Damilola Fajuyigbe, Antony R. Young

**Affiliations:** ^1^ Division of Genetics and Molecular MedicineFaculty of Life Sciences and MedicineSt John's Institute of DermatologyKing's College LondonLondonUK

**Keywords:** skin colour, skin type, photobiology, melanin, ultraviolet radiation

## Abstract

Terrestrial solar ultraviolet radiation (UVR) exerts both beneficial and adverse effects on human skin. Epidemiological studies show a lower incidence of skin cancer in people with pigmented skins compared to fair skins. This is attributed to photoprotection by epidermal melanin, as is the poorer vitamin D status of those with darker skins. We summarize a wide range of photobiological responses across different skin colours including DNA damage and immunosuppression. Some studies show the generally modest photoprotective properties of melanin, but others show little or no effect. DNA photodamage initiates non‐melanoma skin cancer and is reduced by a factor of about 3 in pigmented skin compared with white skin. This suggests that if such a modest reduction in DNA damage can result in the significantly lower skin cancer incidence in black skin, the use of sunscreen protection might be extremely beneficial for susceptible population. Many contradictory results may be explained by protocol differences, including differences in UVR spectra and exposure protocols. We recommend that skin type comparisons be done with solar‐simulated radiation and standard erythema doses or physical doses (J/m^2^) rather than those based solely on clinical endpoints such as minimal erythema dose (MED).

## Introduction

The skin is the body's protective barrier against environmental hazards including solar ultraviolet radiation (UVR). The waveband of terrestrial UVR is ~295–400 nm, of which the majority is UVA (~78% UVA I 340–400 nm; ~17% UVA II 320–340 nm), with UVB (~295–320 nm) typically comprising <5%.

Skin colour is a major human phenotypic trait and has attracted considerable research by many disciplines. The main determinants of skin colour are epidermal melanin pigments, with minor contributions from carotenoids and de/oxyhaemoglobin in dermal capillaries (Alaluf et al., 2001). An understanding of melanin is necessary to determine the role of skin colour in responses to UVR. The lack of solubility of melanin has made its study difficult, but it is known to absorb and scatter across UV, visible and the infrared radiation spectra (Karsten and Smit, 2012).

Much of the evidence for the photoprotective role of melanin comes from the comparative epidemiology of skin cancer. The most recent review on this topic (Brenner and Hearing, 2008) focused on protection against UVR‐induced DNA damage. In this review, we assess the impact of melanin and skin type on a wide range of photobiological outcomes. We also try to identify reasons for discrepancies in outcomes. Our goal is to offer an illustrative rather than extensive summary of the literature and to raise awareness of possible gaps for future study.

## Melanin and skin pigmentation

Skin colour is traditionally defined by the Fitzpatrick skin type system (Fitzpatrick, 1988) that categorizes individuals into six skin phototypes (SPT) (I–VI) based on self‐reported tanning and sunburning susceptibility (Table 1). It is the outcome of a complex polygenic inheritance that involves over 17 genes. The major genes regulating pigment variation span the entire melanogenesis pathway, with MC1R and TYR being the most studied (Beaumont et al., 2009; Dessinioti et al., 2011; Edwards et al., 2010; Gerstenblith et al., 2010; Jagirdar et al., 2014; Parra, 2007). DNA variants in some genes not known to be involved in melanogenesis pathways have also been found to be associated with human pigmentation traits, for example rs12896399 in SLC24A4 (Liu et al., 2013).

**Table 1:** Data have been collected from erythema studies on different skin colours (Fitzpatrick, 1988; European Commision (SCCP), (2006), Harrison and Young, 2002; Godar et al., 2012)

Skin type	Phenotype (non‐exposed site)	Sensitivity to sunburn	1 MED (as SED)	Tanning ability	Susceptibility to skin cancer
I	White (very fair)	Always burns	2–3	Never tans	High
II	White (fair)	Burns easily	2.50–3	Tans minimally	High
III	Cream white	Burns mildly	3–5	Tans gradually	High
IV	Light Brown	Burns slightly	4–7	Tans well	Moderate
V	Brown	Burns rarely	6–20	Tans profusely	Low
VI	Black/dark brown	Never/rarely burns	6–20	Always tans	Low

MEDs are expressed as standard erythema dose (SED) that is independent of personal UVR sensitivity. Note the wide range of SEDs needed for an MED in skin types V and VI, which reflects the uncertainty of the data. Note 1SED = 100 J/m^2^ of UVR that has been biologically weighted with the CIE erythema action spectrum (Diffey et al., 1997).

**Figure 1:**
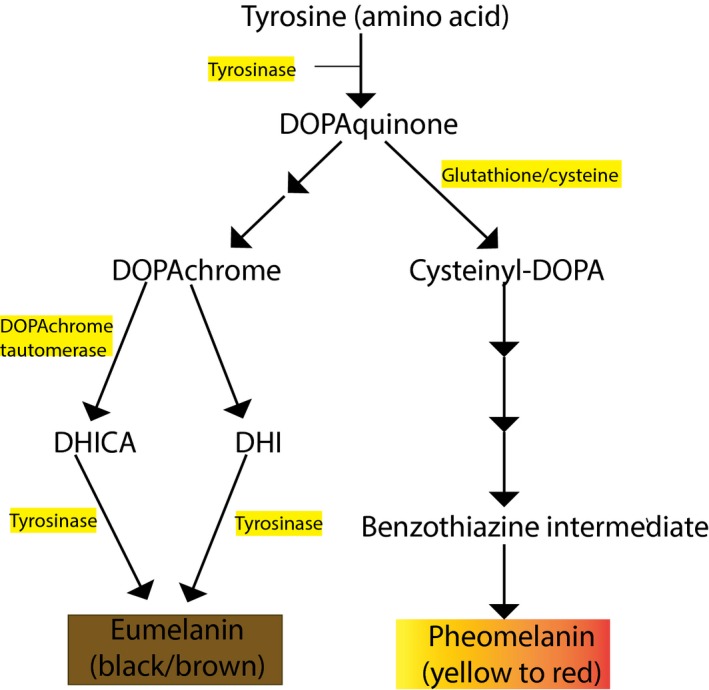
The biosynthesis of the two main melanin pigments in human melanocytes. Downstream of a functional MC1R receptor via αMSH/ACTH stimulation, the black–brown eumelanin is produced, whereas pheomelanin (yellow/red) is derived from the antagonistic action of agouti signalling protein (ASIP) on MC1R in the presence of cysteine (Ito and Wakamatsu, 2008).

Epidermal melanocytes are responsible for the synthesis of melanin (eumelanin and pheomelanin), in lysosome‐like organelles called melanosomes, and its subsequent transfer to adjacent keratinocytes (Figure 1). Within individual melanocytes or keratinocytes, melanin often accumulates as a nuclear cap, which is thought to shield the cells from UVR exposure. Melanocyte number is independent of race but varies with body site (Yamaguchi et al., 2007). Dark skin typically contains four‐ to sixfold more melanin, which is deposited in larger non‐aggregated melanosomes throughout the epidermis, including the outermost *stratum corneum*. In contrast, lighter skin has small aggregated melanosomes, which are restricted to the basal and first suprabasal layers (Alaluf et al., 2001; Brenner and Hearing, 2008).

UVR‐induced melanogenesis, also referred to as tanning, is the skin's response to UVR exposure. It is categorized into three types: immediate pigment darkening (IPD), persistent pigment darkening (PPD) and delayed tanning (DT) (Table 2) (Sklar et al., 2013). IPD presents as a grey‐brownish colour, occurs immediately after UVA exposure, lasts for a maximum of 2 h and then can fade to leave long‐lasting PPD at exposures ≥10 J/cm^2^ UVA. These changes are thought to be the consequence of redistribution, oxidation and polymerization of pre‐existing melanin (Tadokoro et al., 2003, 2005) but neomelanogenesis may also be involved (Wicks et al., 2011). DT is associated with increased melanocyte activity and proliferation. The action spectrum (wavelength dependence) for DT shows a maximum in the UVB region, with a tail in the UVA waveband, and is similar to that for erythema (Parrish et al., 1982) but is very different from the action spectra for IPD and PPD, which show UVA peaks that extend into the visible spectrum (Sklar et al., 2013). It is thought that UVB‐induced tanning offers greater photoprotection against erythema and DNA damage (Coelho et al., 2013) compared to IPD/PPD. This is of concern because the tanning industry promotes sunbeds (mainly UVA I sources) with an implied potential benefit against subsequent UVR exposure.

**Table 2:** Radiation exposure and induced pigmentation. Adapted from Sklar et al. (2013)

Radiation	Pigmentation
UVA	Induces IPD that lasts for a maximum of 2 h and PPD that is longer lasting Delayed tanning appears within 3–5 days after exposure and may persist for months
UVA I	Induces immediate pigmentation and delayed pigmentation in all skin types
UVA II	In skin types I and II, erythema precedes pigmentation In skin types III and IV, it induces IPD with no visible erythema
UVB	Pigmentation occurs when preceded by erythema
Narrowband UVB	Peaks between 3 and 6 days, pigmentation returns to baseline at 1 month
Broadband UVB	Peaks between 4 and 7 days, pigmentation returns to baseline at 3 months
Visible light	IPD and DT; in SPT IV–VI pigmentation may last for 2 weeks

## Effects of terrestrial solar ultraviolet radiation on the skin

### Chromophores

The epidermis is rich in UVR‐absorbing molecules, called chromophores (Young, 1997), many of which have UVR absorption maxima in the non‐terrestrial UVC (100–280 nm) region with tails in the terrestrial UVB and UVA regions. Some chromophores also absorb strongly in the UVA and visible regions. Absorption of UVR/visible radiation energy by chromophores may result in changes in their molecular structures (e.g. DNA, *trans*‐urocanic acid) that directly initiate a wide range of photobiological responses. However, in some cases the chromophore is not the target molecule; for example, energy may be absorbed by chromophores to generate reactive oxygen species (ROS) that damage adjacent molecular and cellular targets. ROS also serves other functions such as in cell signalling. As a rule of thumb, UVB and UVA II cause direct effects in normal skin, whereas UVA I causes indirect effects, via ROS, but there are many exceptions. It should be noted that this rule is largely based on in vitro work.

### DNA photodamage and its consequences

Epidermal DNA is an important chromophore that is very susceptible to structural modification by terrestrial solar UVR, even at suberythemal exposures (Young et al., 1998). The most common DNA lesion is the cyclobutane pyrimidine dimer (CPD) (Premi et al., 2015). Other types of DNA damage include the (6‐4) pyrimidine‐pyrimidone photoproduct (6‐4 PP), Dewar isomers and 8‐oxo‐7,8‐dihydroguanine (8‐oxoGua). The CPD, if unrepaired, may result in characteristic mutations (e.g. C → T transitions) that can lead to skin cancer. There are two major pathways to repair UVR‐induced DNA damage: nucleotide excision repair (NER) and base excision repair (BER). NER is mainly responsible for the repair of 6‐4(PP) and CPD, whereas BER repairs damage to non‐distorting single base modifications caused by oxidation, for example 8‐oxoGua. Inefficient repair is associated with a skin cancer risk that is orders of magnitude greater than normal in xeroderma pigmentosum (XP) patients who have defective NER (Sethi et al., 2013; Wei et al., 1995).

Furthermore, the CPD may trigger non‐genetic effects such as erythema, immunosuppression and photoageing that may increase the risk of skin cancer (Halliday, 2010). Erythema is associated with dermal blood vessel dilation and it is histologically characterized by the presence of sunburn cells (SBCs – apoptotic keratinocytes) 24 h after exposure to UVB and solar‐simulated radiation (SSR). Erythema may be associated with pain, warmth, and reduced mechanical and heat pain thresholds (Harrison et al., 2004). The action spectra for DNA damage (CPD), melanogenesis and erythema are very similar which suggests that DNA is a chromophore for melanogenesis and erythema (Parrish et al., 1982; Young et al., 1998) and indeed erythema may be regarded as a clinical surrogate for DNA damage. UVR also has profound effects on skin immunity (Gibbs et al., 2008) and, for example, antigen‐presenting Langerhans cells (LC) in the epidermis are depleted. Classic experiments in the 1970s showed that UVR‐induced immunosuppression in mice plays a major role in the development of squamous cell carcinoma (SCC) (Fisher and Kripke, 1977; Kripke and Fisher, 1976).

### Photoageing

Photoageing is the superimposition of chronic UVR‐induced damage on intrinsic ageing and may take decades to manifest clinically, characterized by wrinkles, roughness, laxity and uneven pigmentation. These features are associated with degradation of dermal structural proteins such as collagen and elastin by UVR‐induced matrix metalloproteinases (MMP), especially MMP‐1 (Han et al., 2014). Both UVB and UVA I induce a range of MMP at the gene, protein and protein activity level (Fisher et al., 2002; Tewari et al., 2014). It is often stated that ROS induced by UVA is the main cause of photoageing because it penetrates deeper into the dermis; however, we have shown that the action spectra for human erythema and the induction of MMP‐1 gene expression in human skin in vivo are similar (Tewari et al., 2012) which suggests that DNA is an important chromophore for photoageing, as has been proposed by others (Dong et al., 2008) based on in vitro and in vivo studies. Furthermore, photoageing may be linked to SCC. Starcher et al. (1996) found that a neutrophil elastase‐deficient strain of hairless mice was resistant to UVR‐induced photoageing and SCC.

## The role of melanin in photoprotection from the chronic effects of terrestrial solar UVR

### Skin cancer

Melanoma arises from melanocytes, and basal cell carcinoma (BCC) and SCC are keratinocyte‐derived cancers known collectively as non‐melanoma skin cancer (NMSC). The incidence of malignant melanoma (MM) is much lower than that for NMSC, but the former are responsible for the vast majority of skin cancer deaths because they are much more metastatic (American Cancer Society, 2016).

**Figure 2:**
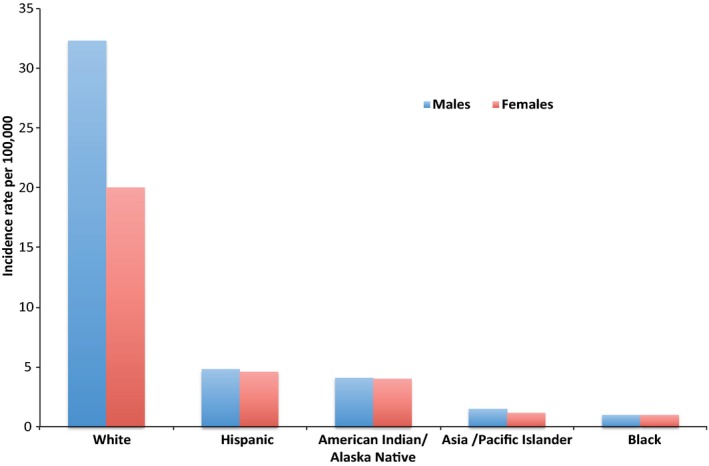
Incidence of Melanoma by race/ethnicity in United States of America, 2007–2011. Incidence rates are from age‐adjusted data (SEER Cancer Statistics Factsheets).

Epidemiological and body site studies have long supported an association between terrestrial solar radiation exposure and all types of skin cancer with the risk of skin cancer being very dependent on skin type/colour. The highest risk of melanoma is in SPT I that has genetically determined phenotypic characteristics associated with greater sensitivity to UVR, including fair skin, light/red hair and eye colour, poor ability to tan and freckling. A USA report showed that the melanoma incidence for white skin males and females is approximately 32 and 20 times higher than their respective black skin counterparts (Figure 2). Reports have also shown a continuous long‐term increase in skin cancer incidence in white populations as opposed to its stable low occurrence in black skin (Gloster and Neal, 2006). Although the incidence of skin cancer is lower in dark skin, the mortality is higher, perhaps a consequence of late diagnosis/misdiagnosis which results in poor prognosis (Bradford, 2009).

The high incidence of actinic damage and NMSC in albinos also suggests a protective role for melanin (Wright et al., 2015). Albinism is a genetically inherited disorder characterized by hypopigmentation of the skin, hair and eyes due to a reduced or lack of cutaneous melanin pigment production. In African albinos, the risk of developing NMSC is up to 1000‐fold greater than the general population (Kiprono et al., 2014) and may present a decade earlier than other patients (Ademiluyi and Ijaduola, 1987).

However, the rarity of melanoma in albinos (even in equatorial Africa) (Lookingbill et al., 1995) suggests that the situation is far more complex. Some authors found that UVR in the presence of melanin can induce ROS (Noonan et al., 2012) and CPD formation (Premi et al., 2015) which was not found in UVA‐irradiated mice lacking melanin. Also, the introduction of an albino tyrosinase (*Tyr* ^*c/c*^ *)* allele into red *Mc1r* ^*e/e*^ background mice protected red mice from melanoma (Mitra et al., 2012) possibly by reducing their pheomelanin content. Individuals with red hair/fair skin (generally with lower melanin content) may have an increased risk of melanoma because of both poor protection from UVR and the photosensitizing potential of pheomelanin (Napolitano et al., 2014).

The role of ‘universally photoprotective melanin’ has also been questioned by observations in vitiligo patients who have areas of depigmented skin. Vitiligo is caused by the local destruction of melanocytes (Nordlund, 2011). An unexpected feature of this condition is the lack of skin cancer on sun‐exposed amelanotic skin. Vitiligo patients do not have a increased risk of NMSC or melanoma compared to the general population, as reported in Tanzanian Africans (n = 76) (Nordlund, 2000) and in Germans with skin phototype (SPT) II/III (n = 136) (Schallreuter et al., 2002) and they may even be at a lower risk (Teulings et al., 2013). The lower probability of NMSC and melanoma may be related to increased levels of wild‐type p53 expression in keratinocytes (Salem et al., 2009; Schallreuter et al., 2003) (which enhances DNA repair ability) and the lack of melanocytes in vitiligo lesions, respectively.

In conclusion, there are factors other than melanin quantity that define skin cancer susceptibility, such as melanin type, DNA repair and antioxidant capacity. Furthermore, personal sun exposure behaviour requires consideration.

### Photoageing

Photoageing affects individuals of all skin colours, but seems to be less pronounced or delayed in those with darker skins (Vermeer et al., 1991). Although there are limited data on photoageing in black skin, and studies are limited to African Americans, the general consensus supports a better preservation of epidermal and dermal components in sun‐exposed black skin in contrast to sun‐exposed white skin (Montagna and Carlisle, 1991), a possible benefit of the reduced UVR transmission in black skin (Kaidbey et al., 1979).

## Experimentally quantifying the photobiological effects of pigmented skin

### Vitamin D

Terrestrial solar UVB radiation is the main source of vitamin D, the synthesis of which is initiated in the epidermis. The chromophore is 7‐dehydrocholesterol, which is photoconverted to previtamin D. A series of thermal and enzymatic processes subsequently result in the biologically active form (1,25(OH)_2_D_3_). Vitamin D is essential for bone health and there are many studies that suggest that it is important for a wide range of health outcomes, although most of the evidence for these has been disputed (Autier et al., 2014). Epidemiology shows that people with pigmented skins typically have lower vitamin D status at given latitude compared with those with lighter skins. A recent systematic review of 12 laboratory studies on the effect melanin on vitamin D (Xiang et al., 2015) concluded that, on balance, pigmented skins are less effective than light skins at vitamin D production, although it should be noted that one recent study by a respected group found no effect of skin type (Bogh et al., 2010) as have others (Lo et al., 1986). Studies from our laboratory (manuscript in preparation) show that melanin reduces the synthesis of vitamin D by a given UVR dose by <twofold, which can be referred to as a protection factor of <2 for this endpoint.

### Erythema

Erythema is the best‐studied response to UVR, especially in fair skin. It is used as an indicator of personal UVR sensitivity by determining the minimal erythema dose (MED), which is the lowest UVR dose necessary for a just perceptible redness (or redness with defined borders) at 24 h post‐exposure (Young et al., 1998). The difficulty of assessing redness on black skin has led to a wide range of MED being reported for black skin (Table 1). The finding that black epidermis is only four times as effective than white skin in attenuating UVB transmission (Kaidbey et al., 1979) suggests two possible explanations: (i) sunburn (assessed by MED) may not be an adequate endpoint to compare skin sensitivity and (ii) melanin's role may extend beyond that of an optical filter of UVR. Both points are addressed below.

### Problems with MED assessment

There are many different methods of assessing erythema, most of which can be confounded in dark skin because of melanin. For example, visual assessment is not only prone to subjective bias but the best that can be done is a semi‐quantitative scale such as +, ++, +++, which discards potentially valuable data. Diffey and Robson (Diffey and Robson, 1992) showed that placing dark plastic filters over white skin increased perceived MED by at least a factor of two. Objective technology such as reflectance spectroscopy is unable to adequately distinguish between the overlapping absorption spectra of haemoglobin and melanin. More recently, UVR‐induced blood flux has been measured by 785 nm laser speckle contrast imaging in different skin types. Skin sensitivity was assessed by the minimum flux dose (MFD), which is in principle similar to the MED (Shih et al., 2015).

### Relationship between MED and Fitzpatrick skin type

Fitzpatrick skin type categories (Table 1) have been widely used as an indicator and predictor of sun sensitivity in epidemiology and experimental photobiology. However, this approach is a subjective and prone to recall error. For example, a study found that only 2/3 people self‐identified as the same skin type after repeat questioning a few months later (Ravnbak, 2010).

Several authors have investigated the relationship between Fitzpatrick skin type and MED (Amblard et al., 1982; Baron et al., 1999; Harrison and Young, 2002; Hemminki et al., 2002; Sayre et al., 1981; Stern and Momtaz, 1984; Westerhof et al., 1990; Wulf et al., 2010; Youn et al., 1997). In general, these studies have shown increased MED with skin type, but with considerable intergroup variation. A typical result is reported in a study of SSR‐induced erythema on the forearm (Westerhof et al., 1990). The mean MED in skin type VI was five times greater than type I but with considerable overlap between the skin types. Overall, the data show that a MED cannot be used to predict an individual's skin type and *vice versa*. A recent study compared the UVB‐induced MED across all skin types with the MFD, which equates to 30% increase in blood flow above baseline. The ratio of mean MED for SPT VI/I was 8.7 but was 6.3 for MFD, supporting the concept of masking by melanin described above. This study also showed much higher coefficients of variation (CV) for the SPT VI MED (87%) compared with MFD (36%) (Shih et al., 2015).

Problems with accurately quantifying MED also arise from the variations in UVR emission spectra used in the assessments. One study (Phan et al., 2006) found a significant relationship (P < 0.001) between MED and skin colour (SPT I‐IV) (n > 69) using SSR (n = 139) and 310 nm UVB (n = 70) but not for 290 nm (n = 69), thus suggesting that basal layer melanin only protects against erythema with wavelengths >290 nm.

The problems discussed above have undoubtedly led to the wide‐ranging UVR doses being defined as the MED in pigmented skin (Table 1). Thus, photobiological comparisons between white and pigmented skin may be unreliable if there is an error in MED assessment. The impact of the large variability in MED assessment for SPT V and VI is also important for phototherapy because dose protocols are often based on MED. An underestimation of MED can unnecessarily prolong treatment time and overestimation causes harm.

In conclusion, many studies show that MED increases with skin type or skin colour, but with considerable overlap between skin types. Indeed, one study comparing MED on vitiligo lesions and adjacent non‐lesional skin of SPT II ‐ VI patients found a maximal difference of about 3 between the two sites. However, the authors also noted a similar ~2.5‐fold skin type‐dependent difference in the MED in the lesional skin which suggests that factors other than melanin content may be important (Caron‐Schreinemachers et al., 2005). Overall, these data suggest that melanin has only a relatively modest effect on MED.

## Melanin protection from acute endpoints relevant for skin cancer

### UVR‐induced DNA damage

DNA photodamage in pigmented and non‐pigmented skins has been compared to determine the photoprotective role of melanin. In some studies, the UVR doses were the same, which makes it relatively easy to calculate a pigment protection factor. However, in others, exposure doses have been based on MED which makes the quantitative assessment of protection by melanin much more difficult, especially if erythema is a clinical surrogate for DNA damage. The problems in the assessment of MED in pigmented skins have been discussed above.

Several studies show an inverse relationship between skin colour and CPD after both similar physical (Del Bino and Bernerd, 2013; Del Bino et al., 2006) and equivalent MED exposures (Bykov et al., 2000; Coelho et al., 2013; Tadokoro et al., 2003). For example, Del Bino et al. (Del Bino and Bernerd, 2013; Del Bino et al., 2006) measured SSR‐induced CPD in ex vivo breast skin in which colour was assessed by reflectance spectroscopy [individual typology angle (^o^ITA)]. Using a range of UVR doses, they found an inverse correlation between ^o^ITA and CPD, including such damage in melanocytes. A beneficial effect of melanin is perhaps further evidenced by the supranuclear localization of melanin in basal layer keratinocytes, which suggests a protective role of the melanin nuclear ‘cap’ (Kobayashi et al., 1998). Furthermore, loading in vitro keratinocytes and melanocytes with melanin reduces the sensitivity to H_2_O_2_‐induced DNA‐strand breaks and cytotoxicity (Hoogduijn et al., 2004; Yohn et al., 1991, 1992).

Skin colour influences the differential distribution of DNA damage in the epidermis. One study (Yamaguchi et al., 2006) found that the amount of DNA damage after 1 MED exposure was similar in the upper and lower epidermis of fair skin, whereas there were at least 1.5‐fold and 2.1‐fold more CPD in the upper epidermis than the lower epidermis in intermediate and dark skin, respectively, at all time points assessed. These results indicate the photoprotective properties of melanin in the basal layer (especially in dark skin). DNA lesions in the basal layer (contains proliferative stem cells) in fair skin may be relevant to the greater susceptibility to skin cancer (Khavari, 2006; Moan et al., 1989).

Assigning a protection factor to melanin is a difficult task, mainly because of the different spectra and dose protocols used in studies. For example, Coelho et al. (2013) exposed skin to 100 mJ/cm^2^ (UVA/UVB) and reported that dark skin/melanin provided a protection factor of around three for CPD, whereas Freeman et al. (1986) reported a dark skin/melanin protection factor >6 when dark and fair skin were exposed to individualized UVB MED. The use of different UVR doses (J/cm^2^), based on MED, with different skin types makes it almost impossible to assign a protection factor for melanin. We advocate the use of the standard erythema dose that is independent of emission spectrum and personal sensitivity to UVR (Harrison and Young, 2002). This will allow for a clearer understanding of the degree of photoprotection offered by melanin.

There are studies that refute photoprotection by melanin pigment in vitro and in vivo (Chalmers et al., 1976; Niggli, 1990; Schothorst et al., 1991). An in vivo study compared CPD after single (0.65 and two MED) and repeated suberythemal (0.65) SSR exposure on buttock skin of SPT II and IV (Sheehan et al., 2002). More damage was seen in skin type IV compared with type II because the former, with higher MED, had higher single and cumulative doses (J/m^2^). Of course, it is possible that this lack of protection may reflect the lack of melanin in previously unexposed buttock skin.

In summary, several in vitro and in vivo studies have assessed the ability of melanin to inhibit DNA photodamage. Some, but not all, have demonstrated protection. The melanin protection factor is typically about 2–3 when UVR doses have been the same. However, this increases to about 6 when doses are based on MED, which is higher in darker skin types. These results are not compatible and are difficult to explain.

### DNA repair capacity

The skin has complex systems to mitigate UVR‐induced damage that include DNA repair mechanisms. Successful repair of UVR‐induced DNA damage relies on the cell cycle arrest at G1/S boundary, which is mediated by the upregulation, and activation of the p53 tumour suppressor protein, otherwise called ‘guardian of the genome’ because of its role in promoting DNA repair and/or apoptotic cell death (Bäckvall et al., 2002). Successful repair may correlate with a strong tanning response via the transcriptional action of p53 on αMSH encoding protein proopiomelanocortin (POMC) (Agar and Young, 2005; Cui et al., 2007; Eller et al., 1994).

In vitro and in vivo (both humans and animal models) found no difference in the rate of DNA repair in different skin colours after acute exposure to similar UVR doses or equivalent MED exposures (Ishikawa et al., 1984; Kobayashi et al., 1993; Schothorst et al., 1991; Smit et al., 2001; Yohn et al., 1992). One study (Tadokoro et al., 2003) found no correlation between epidermal melanin content (fair to dark skin) and removal of CPD at 7 days after MED‐based exposures. This may not be the best time to assess repair differences because the half‐life of CPD (T=T) in those with skin types I/II has been reported at ~20–35 h (Bykov et al., 1999; Young et al., 1996) with very few lesions remaining at 7 days. Furthermore, the physical doses (J/cm^2^) were not comparable, and there is some evidence that repair/apoptosis is influenced by exposure dose (Lisby et al., 2005). Another report showing a lack of correlation between skin colour and DNA repair only investigated Caucasians and thus does not represent the entire range of skin colours (de Winter et al., 2001).

One study (Sheehan et al., 2002) found more CPD in buttock skin of SPT IV after 10 × 0.65 MED, because MED was greater than for SPT II, but there was a significant loss of damage in SPT IV (P = 0.02) after 1 week but not in SPT II (P = 0.18). This suggested that repair was more inducible in the former. Another study found in vitro p53 expression to peak at 24 h in dark melanocytes but to increase steadily up to 48 h in lightly pigmented melanocytes (Barker et al., 1995), thus suggesting that dark melanocytes recover quicker from p53‐induced cell cycle arrest, which suggests that repair was faster. Similarly, the persistent erythema seen in fair skin and some XP patients could correlate with defective DNA repair machinery (Sethi et al., 2013; Wilson et al., 1981). In 2008, a study found a greater frequency of less functional NER proteins ERRC1 and ERRC2, in European Americans (lighter skin) compared to African Americans (dark skin) (Gao et al., 2008).

Furthermore, some studies suggest that MC1R/αMSH regulates DNA repair by increasing production of DNA damage sensors such as ATM, ATR and γHZAX, which promotes quicker assembly of DNA repair proteins (Abdel‐Malek et al., 2014; Swope et al., 2014). Wild‐type MC1R, typical of black skin, but not its red‐hair‐colour variants (typical of SPT I) responded to αMSH with an enhancement of CPD repair (Kadekaro et al., 2010). Similarly, functional MC1R, not its variants, enhances repair of oxidative stress/damage in part by increasing the activity and protein levels of catalase and other antioxidants as well as BER enzymes (Song et al., 2009). As melanoma susceptibility is often related to the presence of ‘loss‐of‐function’ MC1R variants (Maresca et al., 2015), the role of the functional MC1R/αMSH in activating DNA repair suggests that melanin/melanogenesis may have several roles that influence skin cancer risk.

### UVR‐induced apoptosis

Cells with UVR‐induced damage that surpasses their DNA repair capacity either undergo apoptosis, or survive with potentially mutagenic lesions that may result in skin cancer. Apoptotic SBCs can be identified by their histopathology, positive caspase‐3 staining or TdT‐mediated dUTP nick‐end labelling (TUNEL). TUNEL recognizes double‐strand breaks in DNA, a melanin‐mediated mode of cell death (Takeuchi et al., 2004).

The SBC is used as an endpoint in photoprotection studies and the basis of the term ‘biologically effective dose’ (BED). This is the minimal UVR dose that induces one SBC per 0.45 mm length of epidermis. One ex vivo SSR study (Del Bino et al., 2006) reported a linear relationship between skin colour (measured by ^o^ITA) and BED (P < 0.001). The BED in darker skin was approximately two times higher than that in light skin. The lower BED in fair skin was attributed to its higher accumulation of p53 (not serine specific)/unit dose compared to dark skin, although this could also be attributed to photoprotection by melanin.

However, another study (Yamaguchi et al., 2006) assessed SBC after exposing the lower back of different skin types to 1 MED, or similar physical UVR dose (180–200 mJ/cm^2^) using an emission spectrum with 40%UVB/60%UVA. In contrast to Del Bino (Del Bino et al., 2006), they observed SBC colocalized with p53 ser‐46 in dark skin but not in fair skin, as well as a higher level of TUNEL‐positive cells in dark skin compared to light skin. P53 ser‐46 is commonly associated with the apoptotic inducing function of p53. The notion of melanin‐mediated apoptosis is not new; in 1974, Olson et al. (1974) found a greater number of SBC in epidermis containing melanin compared to vitiliginous skin.

The relationship between skin colour and apoptosis is not clear, especially as pheomelanin may play a role (Takeuchi et al., 2004) and conclusive data on the pheomelanin/eumelanin ratios in different skin types are lacking. Furthermore, different UVR exposure protocols, spectra, body sites and methods to quantify skin colour and SBC have been used. It is intriguing that, if dark skin does exhibit increased sensitivity to UVR‐induced apoptosis, its lower accumulation of p53 (as opposed to higher p53 in fair skin) could signify faster DNA repair, and more SBC could signify a more efficient removal of damaged keratinocytes, thus explaining the lower skin cancer incidence in dark skin.

### Photoimmunosuppression

Photocarcinogenesis requires at least two major steps: DNA photodamage, which may lead to mutation, and the suppression of acquired cutaneous cellular immunity. The UVR‐induced suppression of the contact hypersensitivity response (CHS) is widely used in mice and humans as a model of the immunological events in skin cancer (Schwarz and Schwarz, 2011). A normal CHS response can be suppressed if the skin is exposed to UVR before the application of a universal contact sensitizer (hapten). There is good evidence that DNA (CPD) and *trans‐*urocanic acid (*trans‐*UCA), found in high concentrations in the *stratum corneum*, are major chromophores for UVR‐induced immunosuppression.

There are few studies on photoimmunosuppression in different skin types. Some studies have assessed the relationship between cutaneous UCA quantity (Kavanagh et al., 1995; Kral et al., 1967; Snellman et al., 1997; Stäb et al., 1994), rate of UCA isomerization (de Fine Olivarius et al., 1999; Snellman et al., 1997) and erythema susceptibility (de Fine Olivarius et al., 1997), but the results are contradictory. In addition, studies on individuals with a past history of skin cancer and/or healthy individuals have failed to identify a significant difference in the total UCA content (De Simone et al., 2001; de Fine Olivarius et al., 1998) and rate of isomerization between the two groups (De Simone et al., 2001; Snellman et al., 1997).

Three studies have examined the role of skin type on the UVR‐induced suppression of CHS response. One found SPT I/II was more sensitive to suppression of CHS by SSR than SPT III/IV (Kelly et al., 2000) whether exposure dose was quantified by MED or physically. However, previously unexposed buttock skin was used with very little differences in visible pigmentation, which would suggest that factors other than melanin were involved. Selgrade et al. (2001) compared immunosuppressive responses in SPT I‐VI after exposure to a UVB‐rich source. Neither baseline pigmentation nor the tanning response significantly affected the response to UVR‐induced immunosuppression, albeit no graphs or P‐values were given. It appears that the CHS response was dependent on sex, age, UVR dose and batch number of the hapten. Similarly, another study (Vermeer et al., 1991) reported no skin type difference in UVB‐induced immune suppression, and that dark and light skin both exhibited similar losses in Langerhans cell numbers. However, no quantifiable data were given.

It is difficult to draw definitive conclusions from the immunological and CHS studies because of their limited number and methodological variations. One might expect melanin to play a protective role if the chromophore for immunosuppression is basal cell DNA. However, melanin may have a much less important role if suprabasal DNA and/or *stratum corneum* UCA are the important chromophores. Overall, the data on the role of skin type on all photoimmunological responses are inconsistent, with fewer data on skin types V and VI.

### Effect of melanin on biomarkers of photoageing

Although the benefits of melanin in photoageing are exemplified by ‘near‐universal’ occurrence of elastosis in albino patients in Africa (Wright et al., 2015), to our knowledge, there are only two studies on the molecular aspects of photoageing in different skin colours. One study (Fisher et al., 2002) found a greater increase in MMP‐1 mRNA induction (2.6–7) in SPT I/II compared to SPT V/VI after exposure to a suberythemogenic UVA1 dose, and erythemogenic doses (UVB/UVA2 spectrum), despite the higher physical UVR doses being given to those with darker skin. In contrast, Rijken et al. (2004) concluded that an erythemogenic dose is required for SSR‐induced MMP‐1 and MMP‐9 proteins in all skin types studied (SPT I‐III & VI). They exposed the buttocks of subjects to SSR and reported at 24 h post‐exposure no MMP staining at suberythemogenic doses (0.5 MED) but at 2 MED (180–200 mJ/cm^2^) in fair skin (SPT I‐III), a significant increase in the number of MMP‐1 and MMP‐9 protein‐positive cells. In SPT VI, using the same physical dose, no MMP staining was present probably due to the suberythemogenic doses. These studies do not allow any definitive conclusions to be drawn.

## Photoprotection by induced pigmentation

UVR‐induced DT is widely considered to be protective against subsequent UVR exposure; however, the available experimental data suggest it might be of limited benefit.

One study compared DNA damage induced by a 2 MED SSR challenge after 10 consecutive daily exposure of 0.7 MED SSR with and without a UVB sunscreen containing 30 ppm 5‐methoxypsoralen (5‐MOP) which enhances pigmentation (Young et al., 1991). With SSR alone, no protection was observed in SPT I and II, but an induced protection factor (IPF) of about 2 was seen in SPT III‐V. However, slightly greater protection was seen in all skin types after the 5‐MOP treatment. This suggests that a psoralen‐induced tan may afford better photoprotection against DNA damage, but this cannot be recommended, as psoralens are photocarcinogens. Another study (Sheehan et al., 2002) found that, after a cumulative SSR dose of 6.5 MED (10 × 0.65 MED), the induced tan offered a modest IPF of ~2 with no statistically significant difference for protection from both erythema and DNA damage between SPT II and IV. This is similar to IPF reported by Cripps (Cripps, 1981) after assessing protection offered by Wisconsin summer tan by comparing the SSR MED on tanned and untanned skin.

de Winter et al. (2001) exposed participants using a different dose (SSR) fractionation protocol and found that after a cumulative dose of 12.4 MED, the participants (mainly fair skin) showed a decreased sensitivity to erythema by 75% (P < 0.001), equivalent to an IPF of 4.04 ± 0.46, and that CPD formation was reduced on average by 60% (P < 0.0001), equivalent to an IPF of 2.5. Overall, these human studies show that induced pigmentation has limited photoprotection potential that is equivalent to a very low sun protection factor (SPF) sunscreen.

## Conclusions

The powerful evolutionary selection pressures for skin colour have not been definitely established (Elias and Williams, 2013; Jablonski and Chaplin, 2013). Epidemiology suggests that constitutive melanin is extremely effective at preventing skin cancer (with differences in incidence rates at least one order of magnitude) in which case it might be expected to be very effective at preventing DNA photodamage and photoimmunosuppression.

Experimental data show only modest protection against DNA photolesions and erythema (which may be seen as a clinical surrogate for DNA photodamage), either by constitutive or facultative melanin. There are no conclusive data on the ability of melanin to inhibit photoimmunosuppression. Other unknown factors may be important in the differences in skin cancer incidence between black and white skin populations. However, the data on melanin protection of DNA photodamage, if based solely on the limited optical properties of melanin, have profound implications for the prevention of skin cancer in susceptible white skin populations. This is because they suggest that regular use of a low sun protection factor sunscreen or other photoprotection strategies (equivalent to melanin photoprotection – dose reduction factor of 3–6), preferably from an early age, would be very beneficial.

Most photobiology studies have been done on white skin with relatively few contemporaneous comparisons. Often, such comparisons have been done using the MED as the exposure dose unit. Apart from the difficulties in accurately determining an MED in deeply pigmented skin, this means that a skin type VI is typically exposed to a much higher physical dose than a type I. This makes it impossible to calculate the degree of protection afforded by melanin. Furthermore, the action spectra for erythema and DNA photodamage (CPD) are virtually the same, such that CPD is the likely trigger for erythema. This may mean that the endpoint may be predetermined by the exposure dose, all the more so because the CPD may trigger a range of important photobiological responses. Thus, we recommend that physical dose units (J/m^2^) or SEDs are used rather than MEDs. Different studies use different UVR spectra, which also make comparisons difficult. Where possible, we recommend the use of SSR that also accounts for any spectral interactions.
